# Unique Attributes of Guinea Pigs as New Models to Study Ocular Herpes Pathophysiology and Recurrence

**DOI:** 10.1167/iovs.64.14.41

**Published:** 2023-11-28

**Authors:** Tejabhiram Yadavalli, Chandrashekhar Patil, Pankaj Sharma, Ipsita Volety, Hemant Borase, Divya Kapoor, Deepak Shukla

**Affiliations:** 1 Department of Ophthalmology and Visual Sciences, University of Illinois Chicago, Chicago, Illinois, United States; 2 Department of Pathology, University of Illinois Chicago, Chicago, Illinois, United States; 3 Department of Microbiology and Immunology, University of Illinois Chicago, Chicago, Illinois, United States

**Keywords:** herpes simplex keratitis, guinea pigs, rabbits, corneal ulceration

## Abstract

**Purpose:**

The objective of this study was to explore the ocular and systemic outcomes of herpes simplex virus type 1 (HSV-1) infection in guinea pigs, to monitor the spontaneous reactivation of the virus, and to assess the effectiveness of various treatments, drawing comparisons to conventional rabbit models.

**Methods:**

Guinea pigs and rabbits were infected in the right corneas with differing doses and strains of HSV-1. Observations were made over a 71-day period, focusing on comparing ocular lesions, viral shedding patterns, and weight loss between the two animal models. Postinfection, the effectiveness of trifluridine ophthalmic drops, oral acyclovir, and valacyclovir was evaluated. The confirmation of viral infection was done through virus titer assay, fluorescein staining, and corneal imaging.

**Results:**

Guinea pigs and rabbits manifested symptoms akin to human herpes stromal keratitis (HSK) when exposed to varying titers of viral suspension. Regardless of the initial viral load, all guinea pig groups demonstrated comparable ocular pathology, witnessing conditions like blepharitis and conjunctivitis within 3 days, progressing to severe conditions, including total corneal opacification and necrotizing keratitis. Tear film collection revealed nonsignificant differences in viral plaques between all groups. Notably, guinea pigs in the low-infection group experienced the most weight loss, although without significant differences. The replication of the same experiment on rabbits yielded consistent results in disease pathology across different groups, with occurrences of blepharitis and conjunctivitis. Interestingly, after initial resolution, guinea pigs presented a more frequent and broadly observed increase in disease score and corneal opacity, a phenomenon rarely seen in rabbits within the same timeframe. The effectiveness of 1% trifluridine was observed in mitigating ocular HSV-1 disease in both species, whereas oral acyclovir and valacyclovir were found to be detrimental and ineffective in guinea pigs but not in rabbits.

**Conclusions:**

This study demonstrates the potential suitability of guinea pigs as new models for ocular HSV-1 investigations, filling a critical preclinical void of models capable of showcasing spontaneous HSV reactivation in the eye. The observed similarities and differences in the reactions of guinea pigs and rabbits to HSV-1 infection and treatments provide crucial insights, laying the foundation for future studies on ocular HSV pathogenesis, latency, and improved treatment options.

Herpes simplex virus type 1 (HSV-1) is a ubiquitous pathogen^1^ known to cause a variety of diseases in humans, ranging from oromucosal lesions^2^ and cold sores to more severe conditions like keratitis^3^ and viral encephalitis.^4^ Of these, ocular infection leading to keratitis is a major reason for blindness worldwide, with significant associated health care burden. While the infection usually resolves without lasting damage to the cornea, severe and frequent reactivations lead to corneal opacity, scarring, and related pathology. Despite the high prevalence and potential severity of HSV-1 ocular infections, our understanding of the pathogenesis and progression remains incomplete.^5^

Animal models provide a crucial platform to study the pathophysiology of ocular infectious diseases, testing potential therapeutic interventions and establishing the safety and efficacy of new treatments before they go into clinical trials.^6^ In the context of HSV-1 ocular infection, animal models have been instrumental in elucidating mechanisms of viral entry, replication, and host immune evasion and providing valuable insights into viral latency and reactivation. Several studies detail the use of animal models to test antiviral drugs and vaccines. Mouse models are extensively used in the study of HSV-1 ocular infection, in part due to their genetic manipulability and cost-effectiveness.^7^ However, these models have significant limitations when it comes to recapitulating the pathophysiology of the disease in vivo, particularly in terms of corneal scarring and opacity. Size-related constraints of mouse models make it challenging to assess their corneal lesions and recover sufficient amounts of tissue and biosamples for assays—the small tear film volume of mice decreases the efficiency of detecting infectious HSV-1 and HSV-1 DNA. Moreover, mouse models are not effective for studying HSV latency and reactivation events.^8^ These drawbacks are significant as they make it difficult to replicate research findings and pose an obstacle to developing therapies that target both the lytic and latent viral life cycle.

Given these limitations, there is a need for large animal models that allow for better visualization and easier assessment of ocular disease progression and improvement in response to therapy.^9^ One such model that has gained prevalence is the rabbit model. Rabbits offer several advantages over mouse models, including larger eye size for monitoring and gathering biosamples of ocular lesions, and have more similar immune responses to humans compared to mice. However, the use of rabbit models also has its own challenges—a major disadvantage is high cost associated with maintaining and handling these animals, which poses as an accessibility barrier for research groups.^10^

Guinea pigs have been successfully used as models for studying genital herpes simplex virus type 2 (HSV-2) infections and for genital herpes vaccine development.^11^^–^^14^ These animals share many features of genital herpes with humans, including a natural route of inoculation,^15^ self-limiting primary vulvovaginitis,^13^ symptomatic and subclinical shedding of HSV-2, and latent infection of associated sensory ganglia. In an attempt to leverage the strengths of both rabbit and mouse models and generate additional insights into the complex manifestations of ocular HSV-1 disease and responses to standard of care therapies, in this article, we describe the use of guinea pigs as animal models for studying HSV-1 ocular infection and discuss our experimental design, methodology, and preliminary results. We compare the ocular disease manifestation between guinea pigs and rabbits and put forward guinea pigs as the more superior models for studying corneal infection and damage. We also address a gap in research by proposing guinea pigs as effective potential animal models to study viral latency and reactivation as we observed spontaneous viral recurrence episodes.

## Materials and Methods

### Cells

African green monkey fetal kidney epithelial (Vero) cells were a gift from P. Spear (Northwestern University) and grown in Dulbecco's modified Eagle's medium (DMEM) (Thermo Fisher Scientific, Carlsbad, CA) supplemented with 1% Pen/Strep (P/S) and 10% fetal bovine serum.

### Virus

HSV-1 strain McKrae was provided by H. Ghiasi (Cedars Sinai Health System), and HSV-1 strain 17 reporter virus tagged with green fluorescent protein (GFP) was a kind gift from P. O'Hare (Imperial College, London, UK). The viruses were propagated in and titered on Vero cells and stored at −80°C. Viruses were diluted to required strength in PBS and given to the animals on the day of infection.

### Infection Procedure

Guinea pigs and rabbits were intraperitoneally or intramuscularly injected respectively with ketamine/xylazine (45 and 5 mg/kg). Eyes were then treated with 0.5% proparacaine to numb the surface. Guinea pig corneas were subjected to epithelial debridement in a 3 × 3 grid using a 30-gauge needle prior to the application of virus to the scarified region. Virus in a volume of 50 µL was added to the lower conjunctival sac and rubbed three times clockwise and counterclockwise over the cornea to enable infection. Guinea pigs and rabbits received a dose of buprenorphine SR (0.3 mg/kg and 0.15 mg/kg, respectively) at the time of HSV-1 inoculation. Thereafter, rabbits and guinea pigs were provided with oral meloxicam (0.1 mg/kg) starting day 5 postinfection until day 14 daily. As required and based on veterinarian recommendation, buprenorphine ER was also administered every 72 hours to the animals. Animals were assessed for general health, food, and fluid consumption. Humane endpoints included seizures, paralysis, lack of purposeful response to stimuli, periocular self-mutilation, 72 hours of anorexia, progressive deterioration in mentation/obtunded and loss of 20% of body weight, and central nervous system signs consistent with encephalitis. All groups consisted of three animals, minimum numbers required to assess significant differences.

### Drugs

Trifluridine (TFT), acyclovir sodium (ACV), and valacyclovir (VCV) were purchased from the University of Illinois Chicago (UIC) pharmacy. TFT 1% ophthalmic drops were used as is from the dropper bottle. ACV and VCV tablets were pulverized using a mortar pestle in required amounts and mixed with 1% sucrose solution. The drugs were loaded into syringes and stored at room temperature every day prior to use.

### Animals

All animals were purchased from Charles River Laboratories (Wilmington, MA) and housed at the University of Illinois at Chicago Animal Facility (BRL). Experiments involving animals were performed under a UIC-approved protocol (ACC 21-137). All animals were provided water and food ad libitum and housed in a 12-hour light/dark cycle. Both guinea pigs and rabbits were acclimatized to the BRL facility for 1 week prior to the investigators visiting them. The investigators acclimatized themselves to the animals for 1 week and gave the animals sucrose water, carrots (rabbits), and hay (guinea pigs) to help decrease the neophobic nature of the animals. Sucrose water was given to the animals using a rat oral gavage without gavaging the animals. The syringe was placed in the submandibular sac in rabbits and side cheek region of guinea pigs to prevent leakage.

### Virus Titer (Plaque Formation) Assay

Plaque assays were performed as described previously. Briefly, the lysates or supernatants were serially diluted in 10-fold dilutions and added to Vero cells. At 2 hours post infection (hpi), fresh culture medium containing 0.5% methylcellulose was added. Three days later, the cells were fixed with 50% methanol and eventually stained with crystal violet solution. Plaques were counted and quantified.

### Fluorescein Staining

Ophthalmic administration of fluorescein was conducted with a paper strip with one tip stained with fluorescein. The paper strip was moistened with saline water, then placed on the conjunctiva or inferior fornix. After the administration of fluorescein, room light was extinguished to view the fluorescence of the dye under blue light.

### Corneal Imaging

All images shown in this article were procured using an iPhone 14 Pro camera. For fluorescein staining, a fluorescein stick was mixed with 1 mL PBS for 5 minutes, and 5 µL of this solution was dropped on the animal eye. Eyes were artificially blinked two times; excess fluorescein was removed with a cotton arrow. Ambient lighting was switched off, and a blue flashlight (XYSRZ 4 Pack One Mode Blue Light Flashlight; Amazon) was focused on the eyes and photographs were procured. Guinea pigs were not anesthetized during this procedure.

### Corneal Opacity Scoring

Corneal opacity scoring was performed by a blinded reviewer according to the following criteria: 0 = no opacity, completely clear cornea; 1 = slightly hazy, iris and lens visible; 2 = moderately opaque, iris and lens still detectable; 3 = severely opaque, iris and lens hardly visible; and 4 = completely opaque, with no view of iris and lens.

### Statistical Analysis

The data shown in figures are means ± SEMs. The statistical tests were performed on GraphPad Prism version 6.01 (GraphPad Software, La Jolla, CA, USA) and are described in each figure legend. Body weight curves were also generated using GraphPad Prism. Asterisks indicate significant difference: *P < 0.05, **P < 0.01, ***P < 0.001, and ****P < 0.0001.

## Results

### Guinea Pig Corneas Show Ocular HSV-1 Infection Hallmarks and Viral Clearance

**Figure 1.**
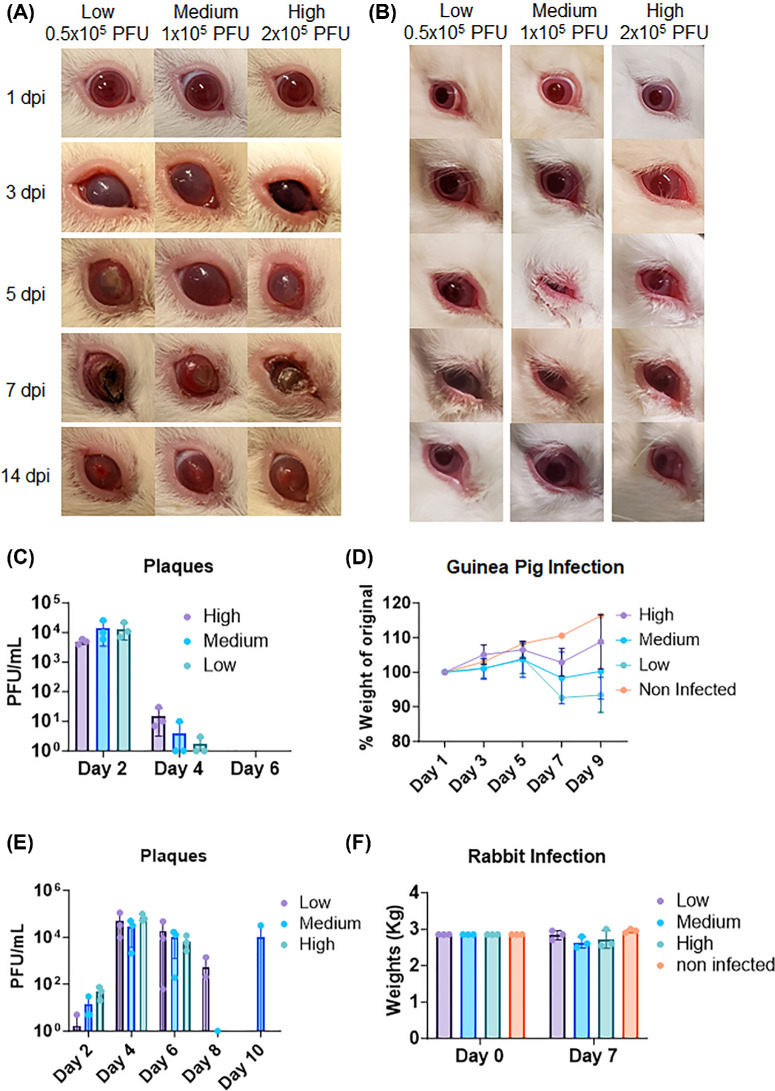
Comparison of ocular HSV-1 infection in guinea pigs and rabbits with different viral doses. (**A**) Photographs of guinea pig eyes at different time points after infection with low (0.5 × 10^5^), medium (1 × 10^5^), and high (2 × 10^5^) titers of HSV-1. (**B**) Number of viral plaques in tear film collected from guinea pig eyes at different time points after infection with different viral doses. (**C**) Weight changes of guinea pigs after infection with different viral doses. (**D**) Photographs of rabbit eyes at different time points after infection with low (0.5 × 10^5^), medium (1 × 10^5^), and high (2 × 10^5^) titers of HSV-1. All three groups showed similar ocular pathology with blepharitis and conjunctivitis. (**E**) Number of viral plaques in tear film collected from rabbit eyes at different time points after infection with different viral doses. (**F**) Weight changes of rabbits after infection with different viral doses. No significant changes were observed during the initial 7 days of infection.

To establish a timeline of the course of HSV-1 ocular infection and observe disease manifestation, guinea pigs were challenged with 0.5 × 10^5^ (low), 1 × 10^5^ (medium), and 2 × 10^5^ (high) titers of viral suspension administered through ocular surface scratch. The animals were subsequently monitored over a course of 14 days for disease progression. Tear film was collected for viral plaque assays every alternate day. Remarkably, all three groups irrespective of the initial viral load added to the eye showed similar ocular pathology with upper-eyelid blepharitis and conjunctivitis seen as early as 3 days postinfection. The corneas pictured as early as day 2 postinfection turned cloudy and progressed to eventual total opacification of the cornea, necrotizing keratitis, and finally open-globe injury (Fig. 1A). Similarly, the number of viral plaques isolated from the ocular washes was nonsignificantly different between all three groups (Fig. 1B). To our surprise, the low-infection group showed the most amount of weight loss between the three groups, although significant differences were not observed (Fig. 1C). To observe the commonality between the two large animal models, we repeated the same experiment in rabbits, another established model for ocular HSV-1 infection for 14 days. The rabbits did not display significantly discernable differences between all three groups in disease pathology, but upper-eyelid blepharitis was observed (Fig. 1D) and conjunctivitis was observed in all three groups. As we did not perform any surgery to remove the third eyelid of the rabbits, procuring open eye images posed a challenge in this model. Unlike mice and guinea pigs, which showed maximum viral load shedding on days 2 and 4, the most amount of viral shedding was seen in rabbits on days 4 and 6 postinfection (Fig. 1E). Like our guinea pig experiment, there were no discernable differences in viral load between the three infection groups, making it an interesting observation. Finally, there were no discernable changes to rabbit weights during the initial 7 days of ocular HSV-1 infection (Fig. 1F).

**Figure 2.**
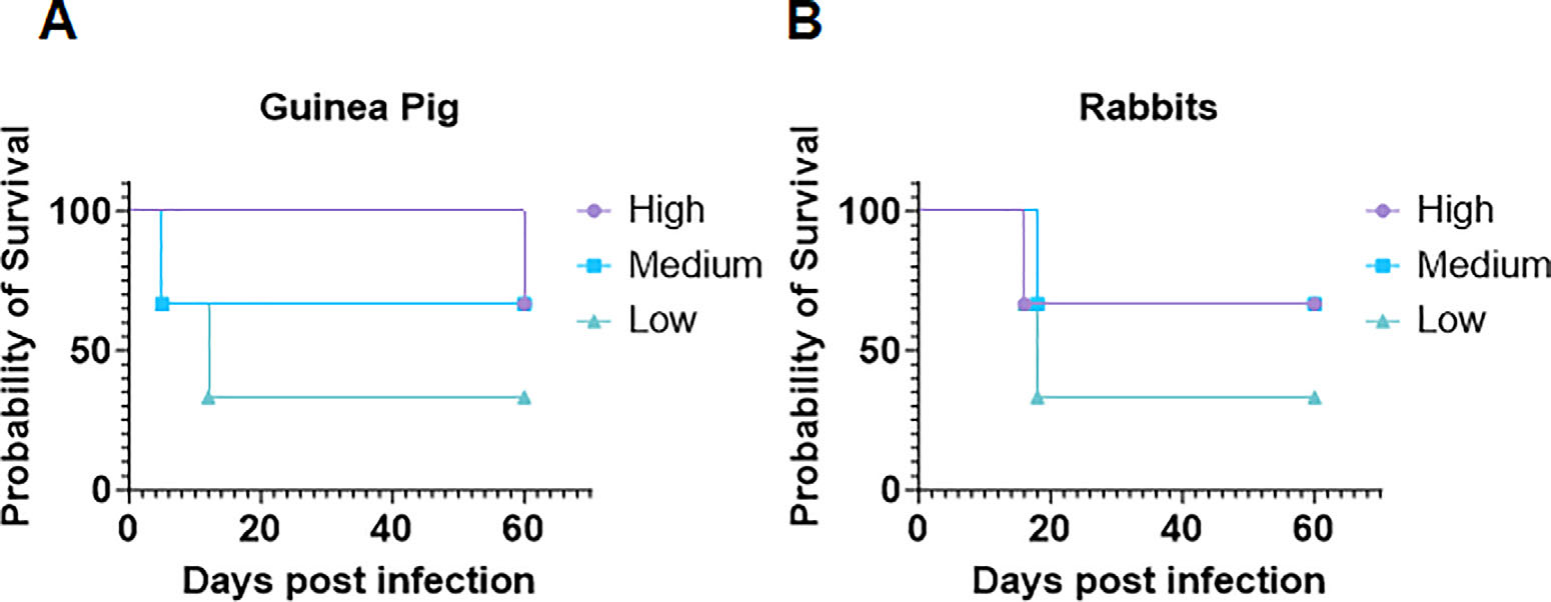
Survival analysis of guinea pigs and rabbits after ocular HSV-1 infection with different viral doses. (**A**) Kaplan–Meier survival curves of guinea pigs infected with low (0.5 × 10^5^), medium (1 × 10^5^), and high (2 × 10^5^) titers of HSV-1. The animals were monitored for 14 days for disease progression and euthanized when they showed severe neurologic symptoms. The low-infection group had the lowest survival rate, followed by the medium and high groups. (**B**) Kaplan–Meier survival curves of rabbits infected with low (0.5 × 10^5^), medium (1 × 10^5^), and high (2 × 10^5^) titers of HSV-1 McKrae.

It is important to note that after the initial 7 days of infection, rabbits and guinea pigs started to show abnormal behavior. Upon consultation with the veterinary group, it was identified that these animals were developing neurologic symptoms, abnormal stool (rabbits), and increased body leaning toward the infected side (right eyes were infected—leaning toward right). While rabbits displayed stereotypical stargazing phenotype^16^ in some cases, guinea pigs displayed lethargic behavior. Based on their weights and severity of the neurologic symptoms, the animals were euthanized. During our experiments, with both rabbits and guinea pigs, we observed a strange coincidence. In both species, we observed that the groups that received the lowest viral load (low group) were one of the first to develop neurologic symptoms severe enough to warrant euthanasia (Figs. 2A, 2B).

### Differences in Disease Pathology Associated With Different HSV-1 Viral Strains in Guinea Pigs

**Figure 3.**
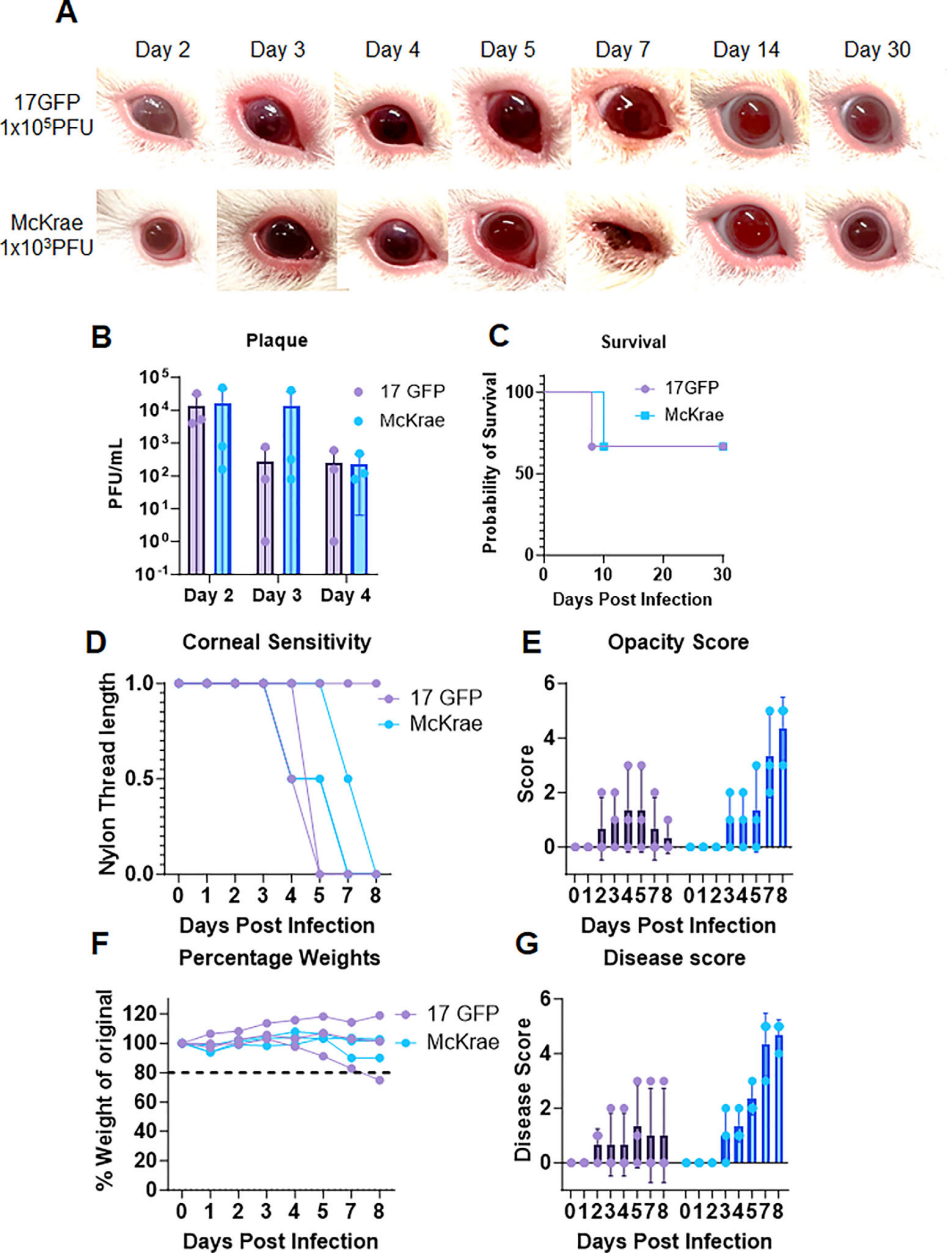
Comparison of ocular HSV-1 infection in guinea pigs with different viral strains. (**A**) Photographs of guinea pig eyes at different time points after infection with HSV-1 McKrae (10^3^) or HSV-1 17GFP (1 × 10^5^). The McKrae-infected group showed more severe ocular pathology than the 17GFP-infected group, with blepharitis, conjunctivitis, and corneal haze. (**B**) Number of viral plaques in tear film collected from guinea pig eyes at different time points after infection with different viral strains. (**C**) Kaplan–Meier survival curves of guinea pigs infected with both viral strains. (**D**) Corneal sensitivity of guinea pigs measured by esthesiometer readings at different time points after infection with different viral strains. (**E**) Corneal opacity scores of guinea pig eyes infected with both viral strains at different time points. (**F**) Weight changes of guinea pigs after infection with different viral strains. (**G**) Disease scores of guinea pig eyes at different time points after infection with different viral strains.

To better understand how low-dose HSV-1 infection caused mortality, guinea pigs were repeat infected with HSV-1 McKrae (10^3^) and fluorescent reporter virus HSV-1 (17GFP) at (1 × 10^5^), which is a less virulent strain, to enable monitoring of disease pathology. The McKrae-infected group showed comparatively rapid disease progression with upper-eyelid blepharitis, conjunctivitis, and low-grade haze. However, guinea pigs infected with 17GFP showed milder pathology compared to McKrae (Figs. 3A, 3E, 3G). Interestingly, viral plaque numbers from both groups were comparable except for nonsignificantly higher numbers in samples collected from the McKrae-infected group, 3 days postinfection (Fig. 3B). However, it was interesting to note that while there was no incidence of whole-globe rupture among the animals in both groups, one member from each group reached endpoint euthanasia criteria. While the 17GFP animal lost greater than 20% body weight, the McKrae-infected animal showed leaning behavior warranting euthanasia (Figs. 3C, 3F).

In our previous studies with mice, we have employed a nylon thread–based esthesiometer reading to determine corneal sensitivity.^17^ While mice responded to a nylon thread length of 6 cm, guinea pigs at basal level did not respond until the thread length was lowered to 1 cm. Our esthesiometer readings showed that both 17GFP and McKrae strains elicited a loss of corneal sensitivity starting day 4 postinfection that never seemed to have returned (Fig. 3D).

### Spontaneous Recurrences of Virus Detected in Guinea Pigs While Absent in Rabbits

**Figure 4.**
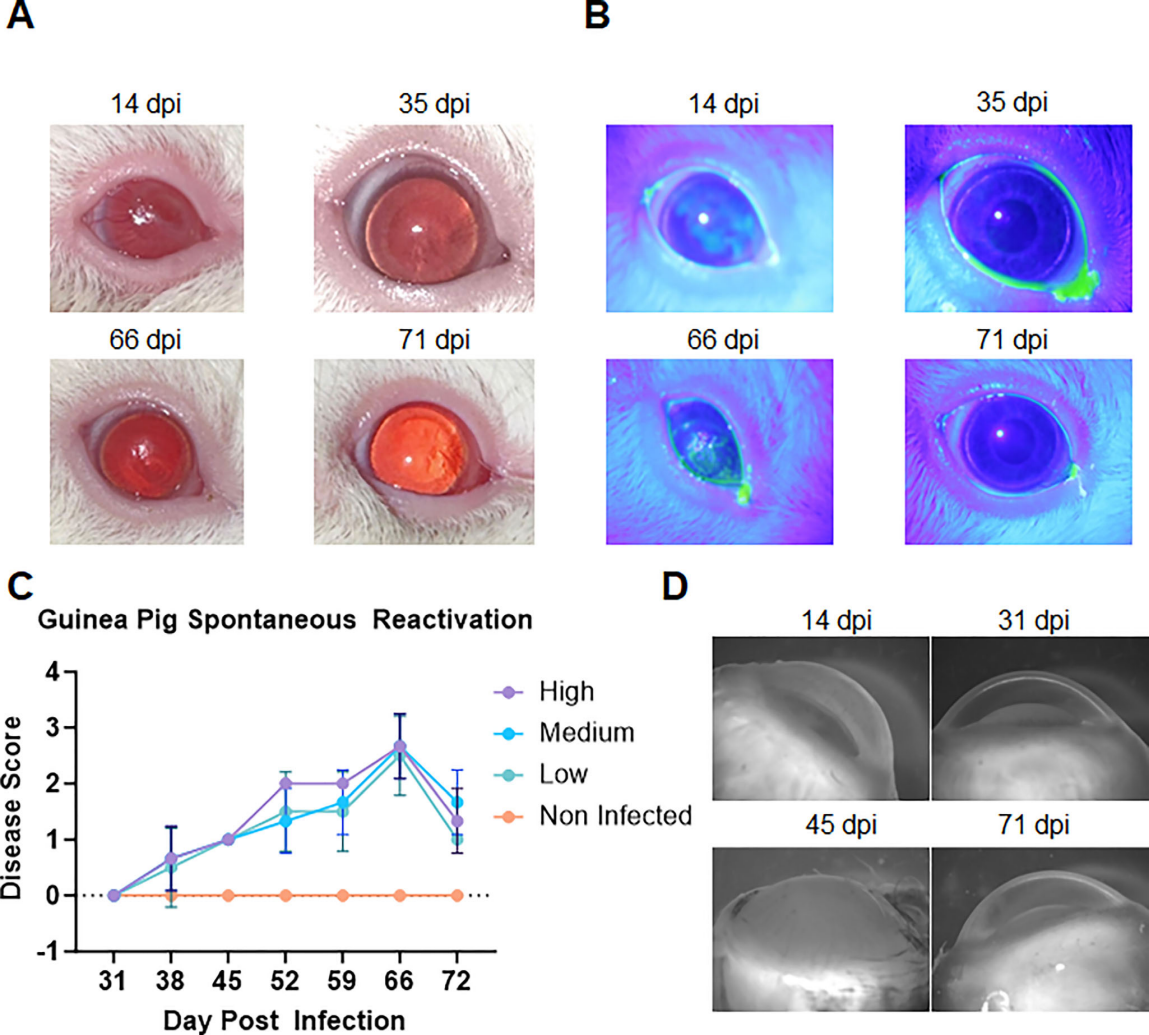
Effect of spontaneous reactivation of ocular HSV-1 infection in guinea pigs. (**A**) Photographs of guinea pig eyes at different time points after infection with low (0.5 × 10^5^), medium (1 × 10^5^), and high (2 × 10^5^) titers of HSV-1 McKrae. The eyes showed corneal inflammation, haze, and scarring that resolved by 30 days postinfection (dpi), but reappeared by day 60 dpi, indicating spontaneous reactivation of the virus. (**B**) Fluorescein stain images of guinea pig eyes at different time points after infection with different viral doses. (**C**) Disease scores of guinea pig eyes at different time points after infection and reactivation with different viral doses. The scores show the increase and decrease in ocular pathology in guinea pigs over a course of 30 days after resolution of initial infection. (**D**) Stereoscope images of guinea pig eyes enucleated after euthanasia at different time points after infection and reactivation with different viral doses. The images show the opacification, resolution, and reopacification of the cornea in HSV-1–infected guinea pigs.

The dearth of spontaneous viral reactivation models for ocular HSV-1 poses a significant obstacle in the development of therapeutics and vaccines. While reports have shown evidence of recurrent infections in genital herpes models of guinea pig, there currently are no studies that discuss the ability of HSV-1 ocular infection to reactivate in guinea pigs. We followed our first set of low, medium, and high viral titer infected animals over a course of 60 days to monitor clearance of disease pathology and tissue inflammation over time. To our delight, we observed spontaneous viral recurrence across all three groups. After 14 days postinfection, most guinea pigs showed a certain level of corneal inflammation, haze, and scarring that completely disappeared in most guinea pigs by day 30, revealing a clear cornea (Figs. 4A, 4B, top row, Supplementary Fig. S1). Starting day 31, we collected ocular washes two times every week to detect any infectious viral dissemination. Unfortunately, we were unable to detect any plaque-forming units (PFUs) from ocular washes collected during this time. Attempts to determine viral load using DNA extracted from the ocular washes also were inconclusive. However, by day 45, we started to observe an increase in the amount of corneal haze in these guinea pigs, which peaked in opacity and scarring by day 60. The opacity and scarring disappeared again by day 70 (Figs. 4A, 4B, bottom row). A quantification of corneal disease score is shown for all groups that shows the increase and decrease in pathology in guinea pigs over a course of 30 days after resolution of initial infection (Fig. 4C). Finally, guinea pig eyes enucleated after euthanasia at different days postinfection were imaged under a stereoscope (Fig. 4D). The images show the opacification, resolution, and reopacification of the cornea in HSV-1–infected guinea pigs. Attempts to observe this in guinea pigs infected with 17GFP and 10^3^ PFU infected McKrae were unsuccessful, suggesting a requirement of higher initial viral load for optimal reactivation.

### Topical Treatments in the Form of 1% TFT Ophthalmic Drops Are Effective in Limiting Ocular HSV-1 Disease and Pathology in Both Rabbits and Guinea Pigs

**Figure 5.**
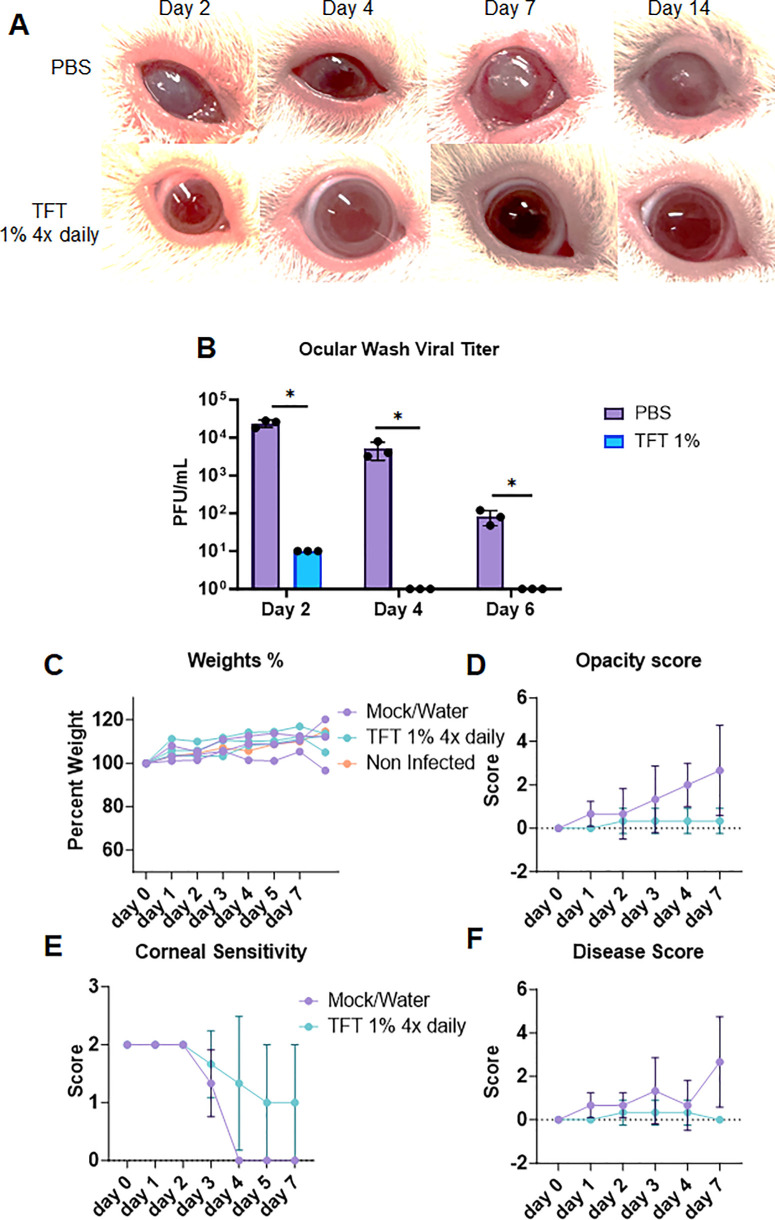
Effect of topical TFT treatment on ocular HSV-1 infection in guinea pigs. (**A**) Photographs of guinea pig eyes at different time points after infection with HSV-1 McKrae. Two groups of animals were infected and treated with either mock (PBS) or 1% TFT four times daily. (**B**) Number of viral plaques in tear film collected from guinea pig eyes at different time points after infection and treatment. (**C**) Weight changes of guinea pigs after infection and treatment. No significant differences were observed between the groups. (**D**) Corneal opacity scores of guinea pig eyes at different time points after infection and treatment. (**E**) Corneal sensitivity of guinea pigs measured by esthesiometer readings at different time points after infection and treatment. (**F**) Disease scores of guinea pig eyes at different time points after infection and treatment. Two-way ANOVA was performed to understand statistical differences between the groups. *P > 0.05.

Given that we were successful in establishing an ocular infection time course with disease symptoms and detectable ocular pathology, we proceeded to testing topical standard-of-care treatments for ocular HSV-1 infection. Two groups of animals were scarified on the cornea and infected with the McKrae strain of HSV-1. Group 1 served as the mock treatment control (PBS administered), and group 2 was administered topical 1% TFT^18^ on the ocular surface, four times daily. Compared to the mock treatment group, the TFT-treated group animals showed minimal to no blepharitis, conjunctivitis, and low/null corneal opacity and visually looked similar to noninfected animal corneas, indicating TFT's efficacy in limiting viral spread and infection and preserving ocular health (Fig. 5A) without losing any weight (Fig. 5C).

The 1% TFT group also showed significantly lower viral plaque numbers over the 6-day course (Fig. 5B). Additional tests, including the corneal opacity scores (Fig. 5D), corneal sensitivity (Fig. 5E), and disease scores (Fig. 5F), also show topical TFT's efficacy in limiting disease-related pathology in guinea pigs.

A comparative study was done in rabbits in parallel to estimate viral loads and disease pathologies. Similar to our guinea pig experiment, rabbits treated with TFT 1% 6 times daily showed minimal to no disease pathology in the form of blepharitis and conjunctivitis (Supplementary Fig. S2). Furthermore, low to no viral plaques were observed in ocular swabs procured from the rabbits treated with TFT.

### Oral ACV and VCV Fail in Constraining Viral Replication and Pathogenesis in Guinea Pigs, Yet Exhibit Efficacy in Rabbits Combatting Ocular HSV-1 Infections

**Figure 6.**
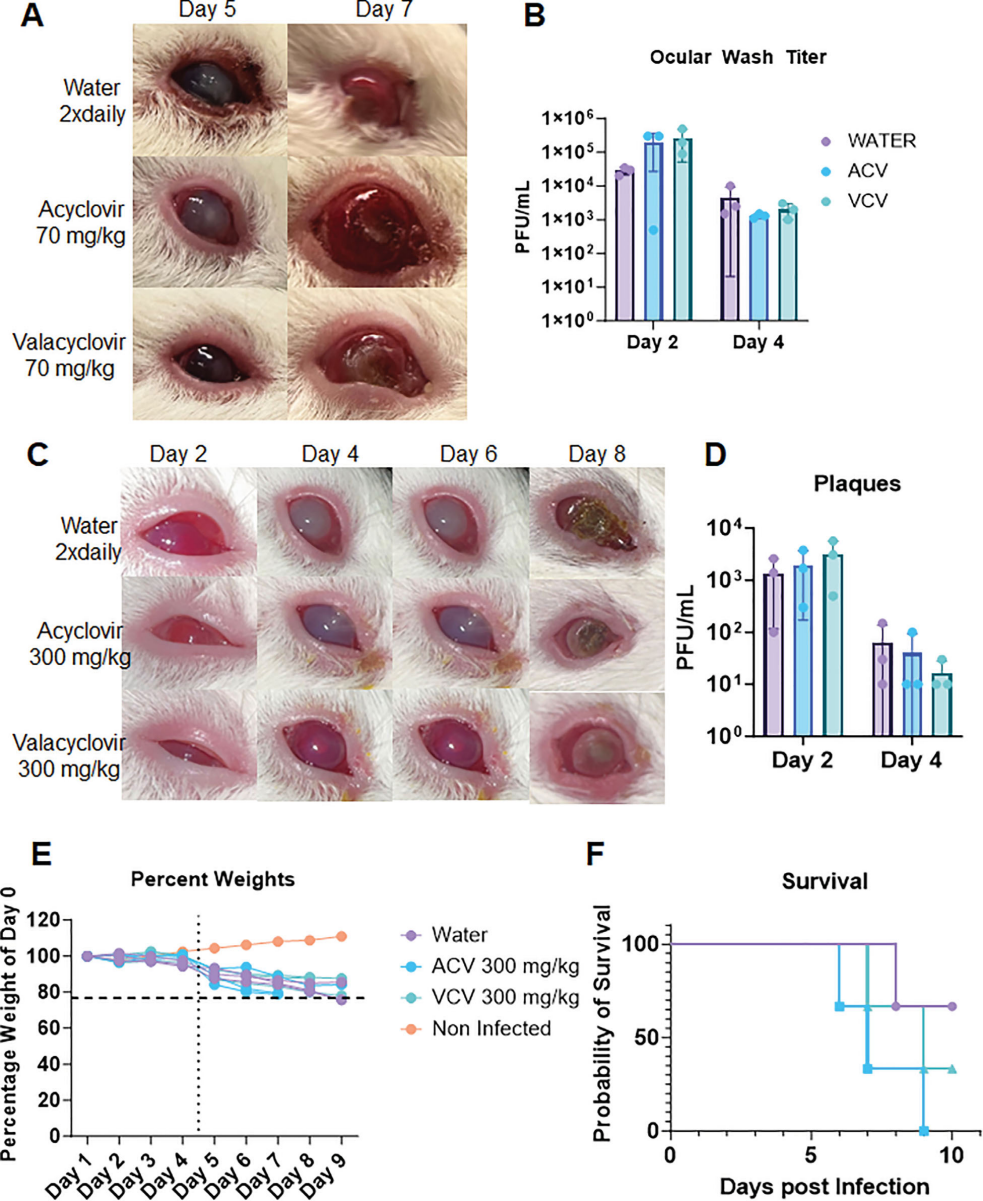
Effect of oral ACV and VCV treatment on ocular HSV-1 infection in guinea pigs. (**A**) Photographs of guinea pig eyes at different time points after infection with HSV-1 McKrae and treatment with mock (PBS), ACV (70 mg/kg), or VCV (70 mg/kg) orally twice daily. (**B**) Number of viral plaques in tear film collected from guinea pig eyes at different time points after infection and treatment. No significant differences were observed between the groups. (**C**) Photographs of guinea pig eyes at different time points after infection with HSV-1 McKrae and treatment with mock (PBS), ACV (300 mg/kg), or VCV (300 mg/kg) orally twice daily. The ACV- and VCV-treated groups showed similar or worse ocular pathology than the mock-treated group, with no improvement in corneal damage. (**D**) Number of viral plaques in tear film collected from guinea pig eyes at different time points after infection and treatment. No significant differences were observed between the groups. (**E**) Weight changes of guinea pigs after infection and treatment. (**F**) Survival curves of guinea pigs after infection and treatment. The ACV- and VCV-treated groups had lower survival rates than the mock-treated group.

Following the success of observing high treatment efficacy to topical 1% TFT, we pursued oral dosages of ACV and VCV (both standard of care)^19^ against ocular disease to generate an understanding of oral treatment options in guinea pigs and their response. Three groups of guinea pigs were used for the experiment: group 1 was a mock treatment, serving as the control; group 2 was ACV administered two times daily through oral gavage at 70 mg/kg; and group 3 was VCV administered two times daily orally at 70 mg/kg. Initially expecting to see effective viral clearance and limited disease spread in both ACV and VCV treatment groups, we were surprised by the low response to ocular treatments and worsening corneal pathology (Fig. 6A) more prominent in the treatment groups. There were also negligible differences in the viral plaque numbers between the mock and ACV/VCV-treated groups (Fig. 6B).

Observing the limitation of 70 mg/kg oral treatment of ACV and VCV, we increased ACV and VCV dosage to 300 mg/kg based on a previous study that used a similar concentration of VCV as a control for their genital herpes study.^20^ To our surprise, we did not observe any observable improvement in disease pathology of ACV- and VCV-treated guinea pigs (Fig. 6C). This was an unexpected finding and maybe linked to a species-specific reaction to these molecules in the face of HSV-1 ocular disease. Further, both treatments did not play a significant role in reducing viral plaques compared to mock treatment (Fig. 6D). Finally, the most astonishing finding was that ACV- and VCV-treated groups succumbed to neurologic symptoms and weight loss (Fig. 6E) earlier than mock-treated controls, warranting their euthanasia (Fig. 6F). These observations, although unexpected, were found by another group studying cytomegalovirus infections in guinea pigs back in 1984.^21^

To confirm whether our findings were due to an issue with the drug formulation, we tested the efficacy of 50 mg/kg valacyclovir two times daily on rabbits infected with ocular HSV-1 (Supplementary Fig. S3). We found excellent activity and minimal disease pathology in VCV-treated rabbits compared to mock controls. We also found a significant decrease in the viral load in treated rabbits when compared to mock-treated controls.

## Discussion

In this study, we introduce guinea pigs as promising models for ocular herpetic diseases, representing a notable shift from their conventional role in studying genital HSV-2 infections and thereby broadening the spectrum of biomedical research. This innovative model uncovers unprecedented avenues for intricate scientific investigations into ocular HSV infections, fostering extensive exploration in the field of ocular infection mechanisms and interventions. The detection of analogous disease pathologies in guinea pigs and humans accentuates the substantial prospects of guinea pigs as pivotal models to decipher the multifaceted nature of ocular HSV infection. This involves delving into how the virus facilitates a prolific infection and induces latency in peripheral neurons, the mechanisms regulating inflammatory eye disease, and the pathways leading to virus reactivation. Gaining insights into these foundational processes is paramount in HSV research, holding paramount significance in propelling the progression of novel treatments and therapies to manage ocular HSV infections effectively.

Our study revealed that the inaugural HSV-1 infection in the guinea pig corneas manifested symptoms mirroring those found in human diseases. These manifestations encompass ulcers in the epithelium, blepharitis, conjunctivitis, epithelial keratitis, stromal keratitis, endotheliitis, and iritis. We recurrently detected spontaneous reactivation of the virus and the resurgence of distinct viral disease symptoms, like corneal dendritic ulcers, substantiating the complications observed in human infections induced by HSV-1. In humans, the primary and ensuing reactivated infections by HSV-1 may present diverse symptoms such as blisters, eye pain, redness, profuse tearing, and, in severe instances, vision loss attributed to corneal scarring. The manifestation of akin symptoms in guinea pigs during both the onset and reactivated infections emphasizes the compelling similarities in disease evolution between guinea pigs and humans afflicted by HSV-1.

Remarkably, our study evidenced spontaneous viral reactivation and the recurrence of viral disease symptoms, including corneal dendritic ulcers, in guinea pigs. This unanticipated discovery highlights the suitability of guinea pigs as potent models for studying ocular HSV infections, elucidating the mechanisms of viral latency establishment and reactivation. Understanding the intricacies of HSV latency and reactivation is a critical frontier in research with monumental implications for developing groundbreaking treatments for HSV infections. Presently, the understanding in this realm is constrained, emphasizing a pressing requirement for animal models that can substantively contribute to bridging this knowledge gap.

Additionally, in the current study, TFT was a successful ocular topical treatment in limiting viral disease and appeared to be nontoxic and well tolerated by guinea pigs. However, standard-of-care oral treatments of ACV and VCV at the tested concentrations (70 mg/kg) failed to yield disease clearance, which was an unexpected finding of the study. When these dosages were increased (300 mg/kg), we observed worsening of corneal health, with significant corneal scarring and opacification. The increased doses of ACV and VCV also appeared to impact animal survival, showing increased mortality. A brief review of literature suggests that both ACV and VCV may not be tolerated well by guinea pigs and may be ineffective as pharmaceutical therapeutics.^21^ Additional testing and future studies would do well to consider this and ascertain alternative modalities. A potential candidate for future testing could be BX795, which has been studied by several groups, including ours, to be well tolerated in vivo and effective in curbing viral infection.^17^^,^^22^^–^^24^ However, a limitation associated with these animals is the lack of validated resources like antibodies to conduct further assays and experiments, which currently impedes the field.

In our current study, we initially assessed viral titers in the tear film of animals exhibiting neurologic symptoms. Subsequently, we collected both trigeminal ganglia (TG) and brain samples to determine the presence and viral load. No virus was detected in brain tissue lysates (data not shown). Additionally, we conducted studies to assess viral titers in the TG, but our findings concerning TG reactivation were inconclusive. It is possible that the virus remains in a latent state within the nerves innervating the cornea, potentially reactivating locally before reaching the TG. While less likely, we cannot rule out the possibility of an alternative site of latency at this point. In the near future, we plan to conduct a more comprehensive study to precisely identify the site of latency in guinea pigs. Furthermore, we are keenly interested in exploring the immune cell landscape and infiltration after HSV-1 infection, as it plays a significant role in driving disease pathology and outcomes. However, we faced challenges in finding suitable reagents, particularly flow antibodies for guinea pigs, to characterize immune cell subpopulations in the current study. In the future, we intend to employ a single cell RNA (sc-RNA) sequencing approach and undertake an extensive study to identify the immune cell populations infiltrating the cornea.

In conclusion, the discovery that guinea pigs can serve as effective models for both HSV-2 genital infection and HSV-1 ocular infection, including spontaneous ocular reactivation, carries significant clinical implications for the field of herpesvirus research and the development of therapeutic interventions. First and foremost, this finding opens new avenues for studying the complex dynamics of HSV infections, shedding light on the mechanisms behind recurrent outbreaks and the factors influencing reactivation. Such insights are invaluable for improving our understanding of the human immune response to herpesviruses and may ultimately lead to the development of more targeted and efficacious antiviral therapies. Furthermore, the guinea pig model's ability to mimic ocular HSV-1 infection and spontaneous reactivation holds promise for advancing research on ocular herpes, which poses a substantial clinical challenge. By studying this phenomenon in a controlled experimental setting, other researchers in the field can explore potential treatment options, especially for therapeutic vaccines, ultimately offering hope for patients with recurrent corneal infections and vision-threatening complications. The use of guinea pigs as models for both HSV-2 genital and HSV-1 ocular infections is an essential finding with far-reaching clinical implications, promising new insights into the pathogenesis and treatment of herpesviruses, particularly those affecting sensitive areas like the eyes and genitals.

## Supplementary Material

Supplement 1
