# Supplementary material for: Unique Attributes of Guinea Pigs as New Models to Study Ocular Herpes Pathophysiology and Recurrence
Source: Invest Ophthalmol Vis Sci. 2023 Nov 28;64(14):41. doi: 10.1167/iovs.64.14.41 (PMC10691389; doi:10.1167/iovs.64.14.41)
Supplement: Supplement 1 [file iovs-64-14-41_s001.pdf]

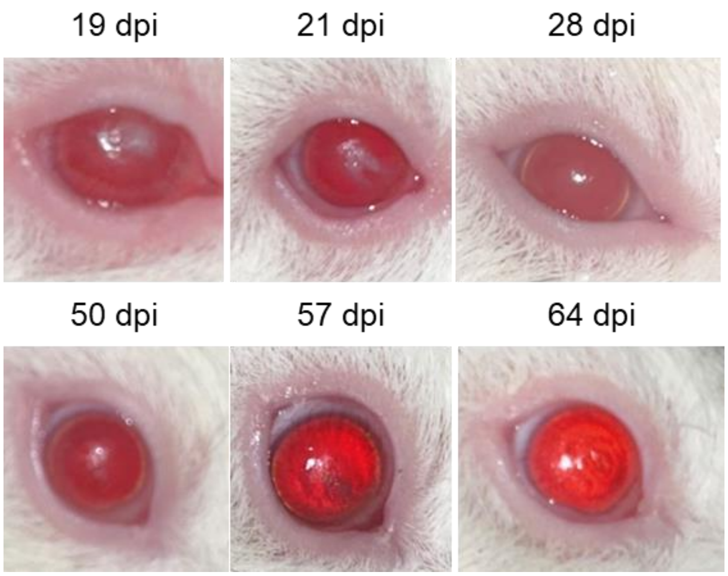

Supplementary Figure 1: Guinea Pig spontaneous Reactivation. Photographs of guinea pig eyes showing resolution, spontaneous reactivation and resolution of ocular disease.

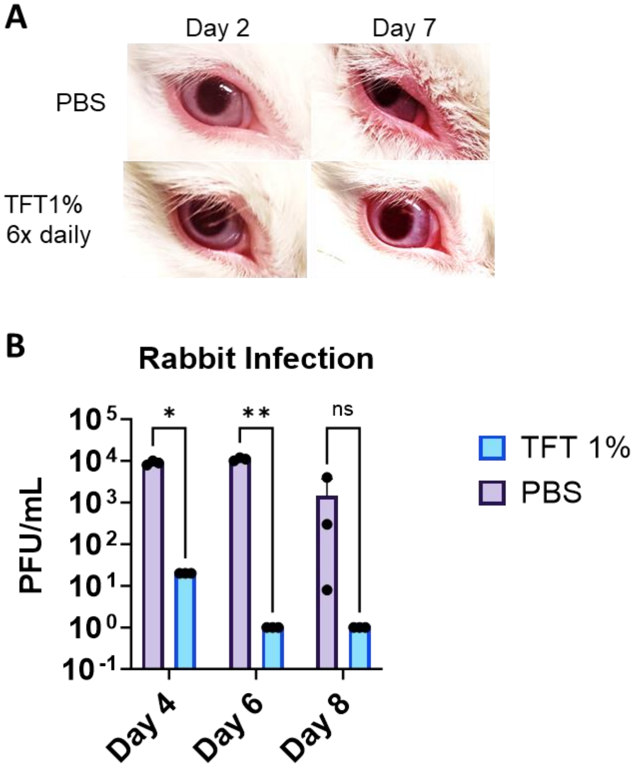

Supplementary Figure 2: Effect of TFT topical treatment in rabbits. (A) Representative rabbit eye images showing disease in HSV-1 McKrae infection treated with PBS or TFT 1%. (B) Ocular swab viral titers determined via Plaque assay. Two-way ANOVA analysis was performed to determine statistical significance. P-value *<0.05, **<0.01, ns-non significant.

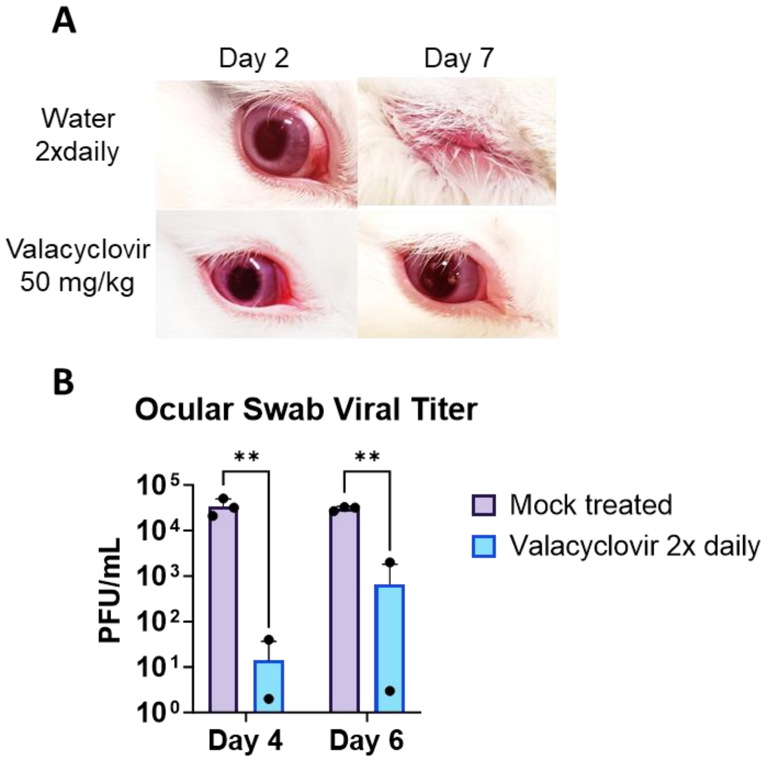

Supplementary Figure 3: Effect of VCV oral treatment in rabbits. (A) Representative rabbit eye images showing disease in HSV-1 McKrae infection treated with VCV 50 mg/kg or water 2x daily (B) Ocular swab viral titers determined via Plaque assay. Two-way ANOVA analysis was performed to determine statistical significance. P-value **<0.01.
